# Mechanical thrombectomy for pulmonary embolism in patients with patent foramen Ovale

**DOI:** 10.1186/s42155-020-00180-9

**Published:** 2020-11-28

**Authors:** Nariman Nezami, Arun Chockalingam, Joshua Cornman-Homonoff, Angelo Marino, Jeffrey Pollak, Hamid Mojibian

**Affiliations:** 1 Section of Interventional Radiology, Department of Radiology and Biomedical Imaging, Yale University School of Medicine, 333 Cedar Street, New Haven, CT 06520 USA; 2 Division of Vascular and Interventional Radiology, Department of Radiology and Radiological Sciences, Johns Hopkins University School of Medicine, Baltimore, MD USA; 3 Department of Radiology, Mount Auburn Hospital, Cambridge, MA USA

**Keywords:** Thrombectomy, Pulmonary embolism, Patent foramen ovale, Anticoagulation, Deep venous thrombosis

## Abstract

**Background:**

The current level of evidence for mechanical thrombectomy (MT) of pulmonary embolism (PE) in patients with patent foramen ovale (PFO) is limited.

**Results:**

This was a retrospective analysis of 9 patients with PFO and acute high-risk or intermediate-high-risk PE, 6 with intermediate-high risk and 3 with high-risk PE. All underwent MT using the Inari FlowTriever System from Dec 2018 to November 2019. Six of these patients had confirmed deep venous thrombosis. The technical and clinical success rate for MT in all patients was 100% and 77.8%, respectively. Right-heart strain improved in 6/8 patients on follow-up echocardiography. Mean main pulmonary artery (MPA) pressure significantly decreased after MT (*p* < 0.012). One patient presented with altered mental status (somnolence and disorientation) prior to coronary artery angiogram and thrombectomy, developed a middle cerebral artery embolic stroke 1 day after MT, and recovered with minor sequalae and later was discharged. There was no in-hospital mortality.

**Conclusions:**

MT using FlowTriever was feasible and safe, successfully improving MPA pressure in patients presenting with concurrent PFO and PE.

## Background

Patent foramen ovale (PFO) in patients with acute pulmonary embolism (PE) can represent a poor prognostic indicator related to risk of paradoxical embolic stroke, which is more common in PE patients with PFO (21.4%) due to increased right ventricle (RV) and atrial pressure resulting in a right-to-left interatrial shunt in the acute phase of PE (Le Moigne et al., 2019).

Standard treatment for hemodynamically stable patients with acute PE is systemic anticoagulation. In acute PE with hemodynamic compromise, the current American College of Chest Physicians (ACCP) recommendation is systemic infusion of thrombolytics over a 2-h period (Saad, 2012). Systemic thrombolysis can be more effective than anticoagulation alone at reducing thrombus burden, but at a price of major bleeding risk of up to 20% (with intracranial hemorrhage accounting for 3–5%) (Saad, 2012). However, if contraindications to thrombolysis, failed thrombolysis, or shock is present, the ACCP suggests a class 2C recommendation for catheter-directed (CDT) thrombolysis. CDT has been shown to have lower risk of bleeding compared to conventional systemic therapy (Kuo et al., 2015), and lower risk of hemorrhagic stroke without change in survival (Liang et al., 2017). Alternatively, percutaneous mechanical thrombectomy (MT) has been showing promise in quickly and effectively treating acute PE. For example, the Inari FlowTriever System (Inari Medical Inc., Irvine, CA), a MT device indicated to treat acute PE, is gaining popularity due to its effective large bore design and ease of use.

While there are multiple case reports on application of catheter based interventions in patients with concurrent PE and PFO, there is no report of MT devices as alternative options in this population (Chockalingam et al., 2020). This case series hence aimed to focus on safety and efficacy of MT using the Inari FlowTriever System in patients with concurrent acute PE and PFO.

## Methods

### Study design and ethics

This was a HIPAA compliant retrospective analysis of 9 patients with acute intermediate-high and high-risk PE for patients with concurrent PFO from two hospital centers affiliated with Yale Medicine from December 2018 to November 2019. This was an investigator-initiated, non-sponsored study, designed to assess the immediate and short-term follow-up outcomes after PA mechanical thrombectomy using the Inari FlowTriever system in patients. This study received an institutional review board (IRB) approval from the Yale School of Medicine implemented in compliance with the Health Insurance Portability and Accountability Act (IRB ID: 2000025511). Obtaining written consent was waived by the IRB committee.

### Study population

All patients within the designated timeframe who were diagnosed with acute intermediate- or high-risk PE, and treated with mechanical thrombectomy using the Inari FlowTriever System were evaluated for concurrent diagnosis of PFO with preprocedural echocardiography.

Patient pulmonary embolism classification as high-risk, intermediate-risk, and low-risk PE were based on the 2011 American Heart Association’s (AHA) Scientific Statement for venous thromboembolism management (Jaff et al., 2011). Intermediate-risk patients were further classified as intermediate-high-risk if hemodynamically stable (Mirambeaux et al., 2019), but showing positive simplified Pulmonary Embolism Severity Index, concomitant echocardiographic RV dysfunction, and positive cardiac troponin. All patients were on anticoagulation without evidence of improvement while deteriorating.

### Imaging

Bedside transthoracic echocardiography was performed on patients by a cardiology fellow or emergency medicine attending with ultrasound training.

Pretreatment CT angiogram of chest was routinely performed for all patients before calling for our institute’s pulmonary embolism response team (PERT) team. A 64 multi–detector row CT scanner (LightSpeed16; GE Medical Systems, Milwaukee, Wisconsin) was used for dynamic CT. Based on PE protocol, PE was defined as any filling defect observed in at least 1 main or lobar pulmonary artery on CT angiogram.

Gray scale, color and spectral Doppler examination of the bilateral lower extremities were performed including the external iliac vein, common femoral vein, deep femoral vein, femoral vein, popliteal vein, tibioperoneal trifurcation, posterior tibial, and peroneal veins. A Philips Ultrasound machine, Model iU22, (Philips Company, WA, USA) with a combination of linear 12 MHZ and curved 5MHZ transducers were used for these exams.

### Mechanical Thrombectomy technique

**Fig. 1:**
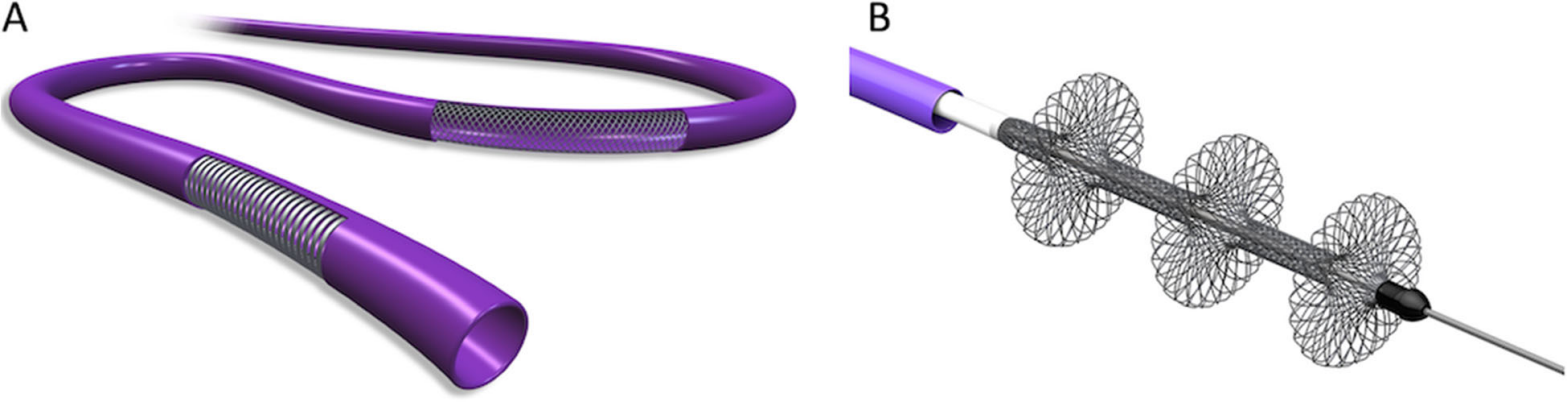
The FlowTriever System. **a** The FlowTriever System consists of a 20Fr/95 cm catheter, called the Triever20, which is placed through the right heart into the pulmonary artery. The Triever20 can be used by itself to perform suction thrombectomy or can be used as a guide through which the FlowTriever catheter is advanced. **b** The FlowTriever catheter has three self-expanding nitinol disks that are unsheathed to engage, disrupt, and extract the thrombus while simultaneously aspirating and withdrawing this through the Triever20

Common femoral vein access was obtained for all 9 patients. A 6 French 100 cm long pigtail catheter was used to measure right atrial and ventricular pressures, perform pulmonary artery angiography, and measure pulmonary artery pressures. A 22 French Gore Dryseal sheath was placed at the access site to accommodate the FlowTriever System. The device is composed of the Triever20 (Fig. 1a) and the FlowTriever catheters (Fig. 1b) (Weinberg et al., 2016; Chauhan et al., 2017).

**Fig. 2:**
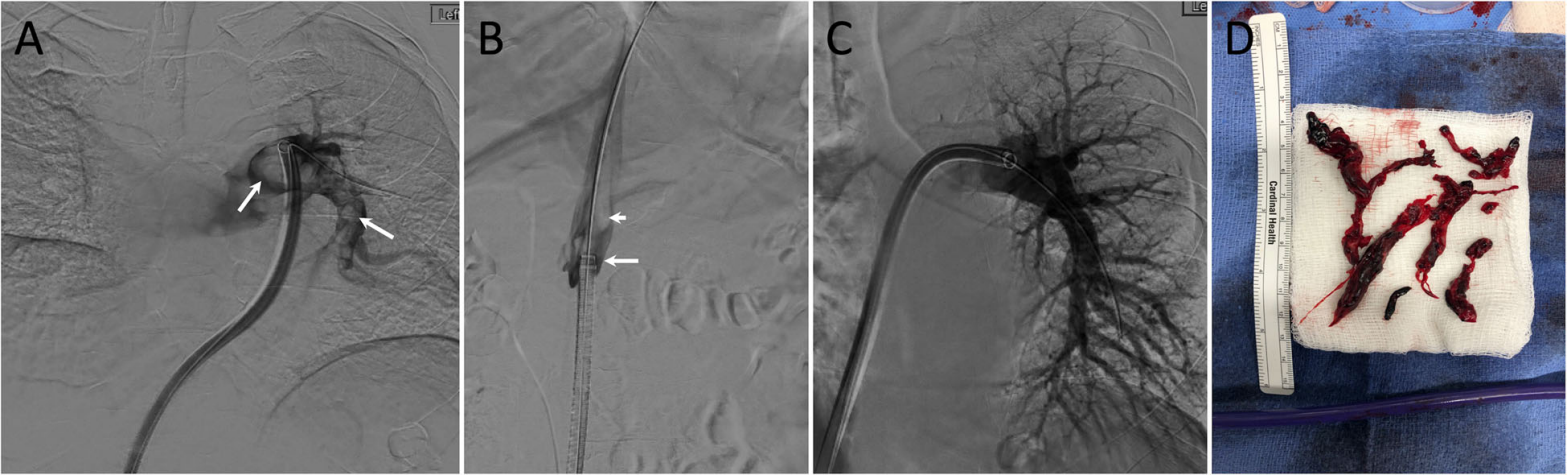
An example of MT in a patient with a clot at the access site, and why there is a concern for paradoxical embolization if the patient has PFO. **a** Initial pulmonary angiogram through the Inari catheter (arrows) shows multiple large filling defects in the left pulmonary artery. The catheter crosses the right atrium, tricuspid valve and right ventricle. **b** Angiogram through a vascular sheath in the right common femoral vein (long white arrow) show large clot (short white arrow) at the tip of the Inari catheter. There is a risk of paradoxical embolization if patient has PFO in case of release of clot from the tip of catheter during catheter pull back. **c** Final angiogram shows almost complete removal of the clots. **d** A sample of thrombus was removed during mechanical thrombectomy

The 20 Fr/95 cm-long Triever20 (formerly known as the Aspiration Guide Catheter) was used for direct clot aspiration via a 60 cc self-locking syringe and was advanced over the guidewire into the right atrium and then to the pulmonary artery under fluoroscopy guidance. Next, suction was applied to retrieve clot from the targeted pulmonary artery, repeated 2 to 3 times. Repeat digital subtraction angiography was performed after clot retrieval to determine the amount of residual clot within the targeted pulmonary artery and demonstrate restoration of blood flow to the distal branches. Repeat PA pressures were measured after the final thrombectomy was complete to assess improvement. In patients with demonstrated deep venous thrombosis in the lower extremity, a cavogram was subsequently performed to look for clot in the inferior vena cava (IVC). If necessary, an IVC filter was deployed in the infrarenal IVC under fluoroscopy. Figure 2 demonstrates an example of MT with visualization of catheter tip and clot.

### Definition of technical and clinical success parameters

Procedure logs, patient demographic data, patient medical history, vital signs, imaging and laboratory variables were collected. Pre- and post-procedural symptoms, stability, pulmonary function, and electrocardiogram (EKG), echocardiographic and CT angiographic findings were documented. Patient outcomes, including major, immediate, or late procedure-related complications and bleeding events were recorded.

Technical success was defined as successful placement of FlowTriever system and initiation of aspiration thrombectomy. An acceptable reduction in thrombus burden was determined intra-procedurally by a minimum of two interventional radiologists on digital subtraction angiograms obtained before and after MT.

Clinical success was defined as meeting all of the following five endpoints: 1) stabilization of hemodynamic parameters (resolution of hemodynamic shock with systolic blood pressure > 90 mmHg without need for vasopressor support); 2) Improved O2 saturation; 3) improvement in pulmonary hypertension 4) Resolution of right sided heart strain; and 5) survival to hospital discharge.

### Statistical analysis

Statistical analysis was performed using SPSS statistical software version 22.0 (IBM Co., Armonk, NY). Pearson’s chi-square test was used to determine patients’ baseline characteristics and clinical variables to determine predictors of adverse outcomes. Nonparametric Wilcox tests were used to compare the effect of mechanical thrombectomy on quantitative variables. A *p*-value less than 0.05 was considered significant.

## Results

Nine patients, five male and four female, had acute PE with concurrent PFO identified on echocardiographic evaluation, and underwent MT. These patients were all on anticoagulation without evidence of improvement and were deteriorating clinically.

The mean age of patients was 67.4 ± 18.6 years. Two patients had prior history of PE and were on anticoagulation. All except one inpatient case first presented to the emergency medicine department. The inpatient had already undergone coronary angiogram on day 1 of admission, after presenting with an altered mental status, chest pain, EKG changes, elevated troponins and concern for myocardial infarction. Her mental status unfortunately continued to deteriorate, and she was deemed a poor surgical and thrombolysis candidate for a newly diagnosed high-risk PE. PE was provoked in 5 patients. Clinical presentation of the patients is listed in Table 1.

**Table 1:** Patient clinical presentations prior to mechanical thrombectomy

Patient	Chest Pain	Dyspnea	Diaphoresis	Dizziness	AMS	Hemodynamics	PE Classification
1	Yes	Yes	No	No	No	Unstable	High-risk
2	Yes	Yes	No	Yes	No	Stable	Intermediate-high-risk
3	Yes	Yes	No	No	Yes	Unstable	High-risk
4	Yes	Yes	No	Yes	No	Unstable	High-risk
5	No	No	Yes	Yes	No	Stable	Intermediate-high-risk
6	No	Yes	No	Yes	No	Stable	Intermediate-high-risk
7	Yes	Yes	No	No	No	Stable	Intermediate-high-risk
8	No	No	No	Yes	No	Stable	Intermediate-high-risk
9	No	Yes	No	No	No	Stable	Intermediate-high-risk

*AMS* altered mental status, *PE* pulmonary embolism

Of these 9 patients, 3 had high-risk PE and the rest had intermediate-high-risk PE. All patients had elevated troponin level, mean and standard deviation of 0.4 ± 0.7 ng/ml. All patients had right heart strain on preprocedural bedside echocardiography. The right-to-left ventricle ratio on CTA was increased in 8 patients, with mean right to left ventricle (RV/LV) ratio of 1.5 ± 0.3. CTA showed bilateral PE in all patients, with a saddle PE in one patient. Eight patients had confirmed deep venous thrombosis (DVT), while one patient’s status was unknown. All patients with lower extremity DVT had an IVC filter placed at the end of the MT procedure.

**Fig. 3:**
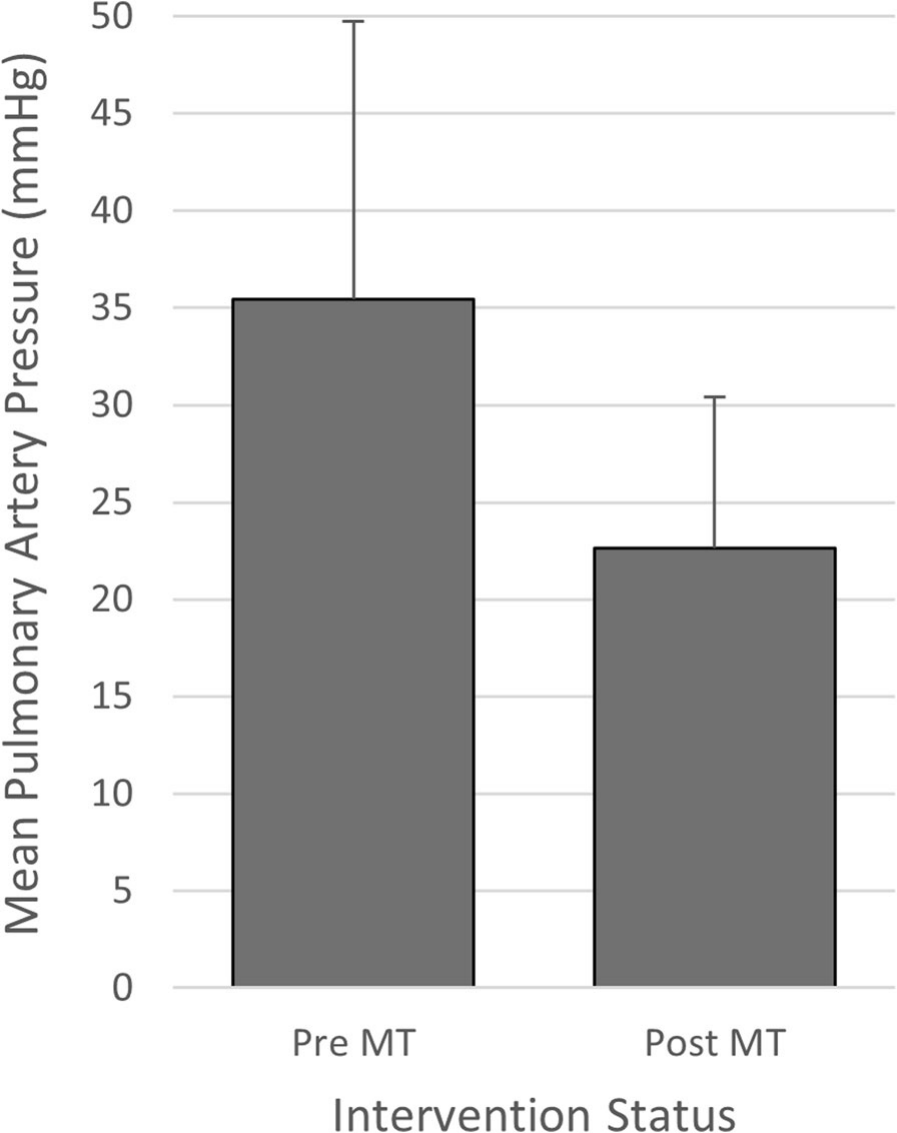
Main pulmonary artery pressure pre and post mechanical thrombectomy (MT). Mean main pulmonary artery (MPA) pressure was significantly decreased after MT (27.7% decrease, *p* < 0.012)

The technical success rate for MT in the 9 patients with concurrent acute PE and PFO was 100%, and the clinical success rate was 77.8%. Right heart strain was improved in 6 of 8 patients on follow-up echocardiography. Persistent right heart strain in one of the patients was attributed to underlying chronic heart disease and in another one to chronic PE based on angiographic findings. Mean main pulmonary artery (MPA) pressure was significantly decreased after MT (36.0 ± 15.2 pre-procedure vs. 23.4 ± 8.4 mmHg post-procedure, *p* < 0.012; Fig. 3). Oxygenation was improved and oxygen requirement was decreased in 7 patients.

The procedure details and outcomes are listed based on the patients in Table 2. There was one patient that was diagnosed with middle cerebral artery embolic stroke 1 day after MT. She initially underwent coronary angiogram on day 1 of admission after presenting with altered mental status, chest pain, EKG changes, elevated troponins and concern for myocardial infarction. Her mental status unfortunately continued to deteriorate, so she was deemed a poor surgical and thrombolysis candidate for newly diagnosed high-risk PE. On day 2 of admission, she underwent successful MT with no immediate complications. She was then diagnosed with middle cerebral artery stroke on day 3 of admission. She recovered from the incident with minor residual speech problems.

**Table 2:** Procedural details and mechanical thrombectomy outcomes

Patient	Access site	Procedure length (min)	Fluoroscopy Time (min)	Post-MT SBP	Post-MT O2 saturation	Change in PA pressure (mmHg)	RHS	Hospitalization (days)
1	LCFV	264	56.6	79	97	−10	No	17
2	RCFV	217	68.9	109	92	−12	No	1
3	RCFV	226	32.1	98	96	−7	No	1
4	RCFV	132	21.7	85	100	−9	No	3
5	RCFV	224	41	116	93	−11	No	3
6	RCFV	151	20.1	176	95	−12	Yes	2
7	RCFV	268	41.9	123	84	− 39	Yes	24
8	LCFV	95	19.4	106	100	−12	No	4
9	RCFV	148	26.7	122	97	−13	No	2

*MT* mechanical thrombectomy, *SBP* systolic blood pressure, *PA* pulmonary artery, *RHS* right heart strain, *LCFV* left common femoral vein, *RCFV* right common femoral vein

## Discussion

MT was feasible and effective in these patients with concurrent acute PE and PFO. There was only one incident of stroke around the time of procedure in a patient who presented to the hospital in an altered mental state, and it is unclear whether the stroke was at all related to the procedure itself.

Successful treatment of PE in patients with PFO has been previously documented in case reports using surgical embolectomy, systemic or catheter-based thrombolysis (Chockalingam et al., 2020; Barbaryan et al., 2019). However, there are no current guidelines for management of PE in PFO patients. Surgical embolectomy has been available for decades for patients in this population who have high-risk PE with contraindications to systemic thrombolysis and has been associated with significant decrease in risk of death and embolization compared to those treated with systemic or catheter-directed thrombolysis (Poterucha et al., 2015). Generally speaking, CDT is favored in poor surgical candidates presenting with hemodynamic instability. However, current literature is limited regarding use of CDT in PFO patients presenting with PE. In patients without PFO, the recent FLARE study (Tu et al., 2019) has shown several advantages to MT: immediate thrombus removal, absence of thrombolytic complications, and reduced need for post-procedural critical care. Possible disadvantages include increased procedural time and the requirement to apply advanced technical skills complications (Saad, 2012). Although patients with PFO presenting with PE are more likely to develop ischemic stroke, presence of PFO is currently not listed as a major or relative contraindication for CDT.

In this study we present the first retrospective experience with this patient population in which MT with the FlowTriever was safe and effective, with the exception of one patient that had an ischemic stroke 1 day after intervention. To our knowledge there has not been even a case report detailing ischemic stroke as a possible complication after CDT. As far as the instance of stroke we report in our study, we are not sure of the mechanism as there is to our knowledge no data published regarding increased risk of paradoxical embolus as a result of catheter-based therapy. The use of MT specifically requires passage of a catheter that could be filled with suctioned clot across the right atrium and ventricle, raising concern for unintentional paradoxical embolism. New thrombus that forms around the catheter could potentially become dislodged during MT and become a source of embolus. These possible mechanisms need to be considered and further investigated as the population of asymptomatic PFO is not insignificant.

There are a few limitations to our study. While this is the first report on application of mechanical thrombectomy using Inari FlowTriever device in patients with PFO on transthoracic echocardiography, we might have missed patients with small PFO in the rest of our retrospective population. Although we evaluated for PFO using transthoracic echocardiography, transesophageal echocardiography with bubble study has been shown to have higher specificity (Doyen et al., 2014). We suggest obtaining a transesophageal echocardiogram with bubble study before MT, in addition to screening for DVT particularly at the access site and assessing IVC clot via cavogram. If thrombosis of one of the femoral veins is present, we recommend accessing the other side. In the presence of a large PFO, reconsidering MT and referring to possible surgical embolectomy is an option. Concurrent clot in the right atrium, right ventricle or across the PFO on CT chest angiogram could make the patient a surgical candidate. A larger prospective study could provide more information on the true efficacy of MT in this specific population.

## Conclusion

CDT for patients with PFO suffering from acute PE could be successfully performed, though more substantial level of evidence is still required to validate this approach as standard of care in high-risk patients. Case series such as ours could provide the foundation to strengthen the body of evidence to hopefully improve quality of treatment, especially for patients with PFO.

## Data Availability

Available for review.
